# “Lipid Nutrition Guidelines: A Comprehensive Analysis” by Harumi Okuyama, Sheriff Sultan, Naoki Ohara, Tomohito Hamazaki, Peter H. Langsjoen, Rokuro Hama, Yoichi Ogushi, Tetsuyuki Kobayashi, Shunji Natori, Hajime Uchino, Yoko Hashimoto, Shiro Watanabe, Kenjiro Tatematsu, Daisuke Miyazawa, Mikio Nakamura and Kentaro Oh-hashi

**DOI:** 10.3390/nu13082828

**Published:** 2021-08-18

**Authors:** Artemis P. Simopoulos

**Affiliations:** Center for Genetics, Nutrition and Health, Washington, DC 20016, USA; cgnh@bellatlantic.net

This book, “Lipid Nutrition Guidelines: A Comprehensive Analysis” by Professor Harumi Okuyama et al. is very timely. Professor Okuyama, the senior author, along with 16 prominent scientists have contributed many outstanding papers in the field over the years. The book consists of four chapters, an epilogue, key issues, notes added in proof and 174 references.

Each chapter ends with a summary and includes recommendations. Tables and Figures are clear and well described. Although the cited literature is international as it suits this field, many of the issues, in terms of actions taken, come from recommendations of the various professional and governmental groups in Japan with which the authors disagree.

Chapter 1. How the Cholesterol Hypothesis Lost Credibility: A Brief Summary.

The authors provide evidence on what has been wrong with the cholesterol hypothesis “Low density lipoprotein (LDL) cholesterol in causing CVD.” Although LDL continues to dominate the field as causing CVD, it has never been proven that low LDL prevents cardiovascular disease, and many patients who die from cardiovascular disease indeed have low LDL.

The authors critique of the various meta-analyses is exemplary, and the reader will learn much about the misconceptions and errors in the data interpretations. The first chapter focuses on the metabolism of the n-6 and n-3 and the importance of a balanced n-6/n-3 ratio. Professor Okuyama is a pioneer in the n-6/n-3 field and the importance of the balanced n-6/n-3 ratio, as was the late doctor Professor Hamazaki, to whom the book is dedicated. These authors present a precise critique of the American Heart Association’s (AHA) interpretation for recommending increased intake of vegetable oils that led to a high n-6/n-3 ratio [1].

In Chapter 2 “All Mainstream Lipid Nutrition Guidelines Disregard Evidence That Some Types of Vegetable Fats and Oils Induce Stroke and Disrupt Endocrines,” the authors discuss the studies and provide experimental evidence that vegetable oils, especially Canola, high in erucic acid, and hydrogenated soybean oil, are “toxic” and detrimental to health. Most of these studies are carried out in the Stroke-Prone Spontaneously Hypertensive Rat (SHRSP) and may not be transferable to humans as such. In fact, the authors admit to that. There are interesting descriptions from both animal and human studies on the role of Vitamin K1 (Dihydro-VK1) in addition to trans fatty acids and VK2-Osteocalcin Link in the prevention of CVD, about which there has not been much discussion by the Committees that develop Dietary Guidelines either at the country level or the World Health Organization (WHO) level.

Chapter 3. Industry-Oriented Lipid Nutritional Guidelines Have Likely Endangered Some Populations.

This chapter is focused on changes in dietary patterns that occurred in Japan following dietary recommendations to increase vegetable oils, primarily n-6 oils, that led Professor Okuyama to describe “the n-3 deficiency syndrome” [2]. The authors focus on the role of vegetable oils as endocrine disruptors in affecting sexual development and mental disorders and recommend that “additional data must be collected on the intake of different types of fats and oils in different countries and ethnic groups”. The authors question the health aspects of olive oil. This is a controversial area, and the authors will have to come up with better data than the animal model of the SHRSP. Additionally, all olive oils are not the same either in the fatty acid composition or antioxidant content.

Chapter 4 is on “Comprehensive Risk Management in Light of Japan’s Medical Care Act.” The authors make a point that many of the decisions have not been based on appropriately conducted clinical trials either using nutrients or drug interventions. This concern has also been expressed by Nissen relative to the 2015–2020 Dietary Guidelines of Americans [3].

In conclusion, this book by Professor Harumi Okuyama et al. should serve as a stimulus for other countries to evaluate their dietary guidelines considering that there are ethnic differences. Genetic differences and the genetic variants of familial hypercholesterolemia in causing CVD should be studied separately than the rest of the population’s cholesterol levels by each country. This is indeed the time to move forward, particularly as the NIH established the “All of Us Program” [4] in 1918 and “Precision. Nutrition Research Program” in April 2021 [5]. The time has come to move away from hypotheses that have not been proven and make decisions based on association studies. Genetic variation in populations and the role of Nutrients in gene expression in human studies will correct the inadequacies of current Dietary Recommendations and will limit the interferences of the Food and Pharmaceutical industries.

In the meantime, the book should be of interest to those interested in Nutrition Research in general and professionals in the fields of Cardiology, Genetics, Immunology, Dietetics, Food Technology, Food Sciences, Agriculture and in Nutrition Policy.

## Data Availability

Not applicable.
